# TGF-β1 Provides Neuroprotection via Inhibition of Microglial Activation in 3-Acetylpyridine-Induced Cerebellar Ataxia Model Rats

**DOI:** 10.3389/fnins.2020.00187

**Published:** 2020-03-20

**Authors:** Bei-Bei Cao, Xiao-Xian Zhang, Chen-Yu Du, Zhan Liu, Yi-Hua Qiu, Yu-Ping Peng

**Affiliations:** ^1^Department of Physiology, School of Medicine, Nantong University, Nantong, China; ^2^Co-innovation Center of Neuroregeneration, Nantong University, Nantong, China

**Keywords:** TGF-β1, neuroinflammation, cerebellar ataxia, microglia, neurodegeneration

## Abstract

Cerebellar ataxias (CAs) consist of a heterogeneous group of neurodegenerative diseases hallmarked by motor deficits and deterioration of the cerebellum and its associated circuitries. Neuroinflammatory responses are present in CA brain, but how neuroinflammation may contribute to CA pathogenesis remain unresolved. Here, we investigate whether transforming growth factor (TGF)-β1, which possesses anti-inflammatory and neuroprotective properties, can ameliorate the microglia-mediated neuroinflammation and thereby alleviate neurodegeneration in CA. In the current study, we administered TGF-β1 via the intracerebroventricle (ICV) in CA model rats, by intraperitoneal injection of 3-acetylpyridine (3-AP), to reveal the neuroprotective role of TGF-β1. The TGF-β1 administration after 3-AP injection ameliorated motor impairments and reduced the calbindin-positive neuron loss and apoptosis in the brain stem and cerebellum. Meanwhile, 3-AP induced microglial activation and inflammatory responses *in vivo*, which were determined by morphological alteration and an increase in expression of CD11b, enhancement of percentage of CD40 + and CD86 + microglial cells, upregulation of pro-inflammatory mediators, tumor necrosis factor (TNF)-α and interleukin (IL)-1β, and a downregulation of neurotrophic factor, insulin-like growth factor (IGF)-1 in the brain stem and cerebellum. TGF-β1 treatment significantly prevented all the changes caused by 3-AP. In addition, *in vitro* experiments, TGF-β1 directly attenuated 3-AP-induced microglial activation and inflammatory responses in primary cultures. Purkinje cell exposure to supernatants of primary microglia that had been treated with TGF-β1 reduced neuronal loss and apoptosis induced by 3-AP-treated microglial supernatants. Furthermore, the protective effect was similar to those treated with TNF-α-neutralizing antibody. These findings suggest that TGF-β1 protects against neurodegeneration in 3-AP-induced CA rats via inhibiting microglial activation and at least partly TNF-α release.

## Introduction

Cerebellar ataxias (CAs) comprise a heterogeneous group of neurodegenerative diseases hallmarked by dysfunction of the cerebellum along with a host of neurological symptoms (Brusse et al., 2007; Marmolino and Manto, 2010). The most common clinical manifestations related to CA include gait abnormality, cerebellar atrophy, and Purkinje cell degeneration (Tatsuoka et al., 1985; Marsden and Harris, 2011; Marsden, 2018). Accumulating evidence has shown that neuroinflammation, the inflammation of the central nervous system, constitutes a fundamental process involved in the progression of neurodegenerative diseases, such as Alzheimer’s disease (AD), Parkinson’s disease (PD), amyotrophic lateral sclerosis (ALS), Huntington disease (HD), multiple sclerosis (MS), and spinocerebellar ataxia (SCA) (Chen et al., 2000; Platten and Steinman, 2005; Wang et al., 2005; El Khoury et al., 2007; Glass et al., 2010; Liao et al., 2012; Cvetanovic et al., 2015). Neuroinflammation is generally regarded as a result of activation of glial cells including microglia and astrocytes (Gogoleva et al., 2019), in which microglia, the primary mediators of neuroinflammation, can produce cytotoxic compounds, cytokines and chemokines, which are capable of inducing neuronal damage in different and complementary ways. Importantly, a few of reports suggest the existence of activated glial cells in CA brain. For example, in a mouse model of SCA1, astrocytes and microglia are activated very early in disease pathogenesis even in the absence of neuronal death and glial activation closely correlates with SCA1 progression with the development of glia-based biomarkers to follow disease progression (Cvetanovic et al., 2015). Moreover, using PLX, an inhibitor of colony-stimulating factor 1 receptor (CSFR1), to deplete microglia at the early stage of disease results in the amelioration of motor deficits in SCA1 mice (Qu et al., 2017). Additionally, the results from proton magnetic resonance spectroscopy research indicate that gliosis is closely related to disease severity in SCA1 patients (Oz et al., 2010). These findings demonstrate that glia-mediated neuroinflammation actively contributes to the pathogenesis of CA.

Transforming growth factor (TGF)-β1, belonging to the TGF-beta superfamily including three isoforms (TGF-β1, 2, and 3) in mammals, is a pleiotropic cytokine that regulates cell survival, inflammation, differentiation and apoptosis (Taipale et al., 1998; ten Dijke and Hill, 2004; Pál et al., 2012), and also exerts a central role in immune suppression, and repair after injury (Li M. O. et al., 2006). TGF-β1 is generally thought to be protective against neuronal damage induced by hypoxia/ischemia, excitotoxins and tropic factor deprivation (Vivien and Ali, 2006). Recently, the results from our laboratory have suggested that TGF-β1, with its immunosuppressive and anti-inflammatory properties, plays a neuroprotective role in neurodegenerative diseases by inhibiting the process of neuroinflammation. For instance, in AD model rats, TGF-β1 pretreatment remarkably ameliorates amyloid-β (Aβ)_1__–__42_-induced neurodegeneration and prevents Aβ_1__–__42_-induced increases in glia-derived proinflammatory mediators and T cell-derived proinflammatory cytokines, in the hypothalamus, serum or cerebrospinal fluid (Chen et al., 2015). Besides, the TGF-β1 administration after the Aβ_1__–__42_ injection ameliorates cognitive deficit and neuronal loss and apoptosis, reduces amyloid precursor protein expression, elevates protein phosphatase (PP)2A expression, attenuates glial activation and alleviates the imbalance of the pro-inflammatory/anti-inflammatory responses of T-lymphocytes (Shen et al., 2014). Furthermore, in AD cell model, TGF-β1 protects neurons against Aβ_1__–__42_-induced neuronal inflammation and apoptosis by the activation of transforming growth factor-β receptor type 1 (TβR-1) (Fang et al., 2018). In addition, we have also found similar results in PD that TGF-β1 plays neuroprotective role by inhibiting microglial inflammatory responses via activating TβR-1 and its downstream signaling pathways in microglia (Liu et al., 2016; Chen et al., 2017). CAs also belong to neurodegenerative diseases, however, the role of TGF-β1 in CA neuropathology remains elusive. Therefore, we were interested in whether TGF-β1 can play a protective role in CA and the particular mechanism involved in it was worthy of our attention.

Neurotoxin 3-acetylpyridine (3-AP), an antimetabolite of nicotinamide, specifically damages calbindin-expressing neurons in the inferior olive, which sends axons, termed climbing fibers, to form synapses on Purkinje cells (Wecker et al., 2017). Destruction of the nerve fibers innervating the cerebellum by 3-AP can induce CA and provoke inflammatory reactivity present in animal brain in CA (Riva-Depaty et al., 1994). Thus, in the present study, we administered 3-AP by intraperitoneal injection in rats to induce CA-associated changes *in vivo*, and focused on the anti-inflammatory property of TGF-β1 by determining microglial functions so as to exhibit its neuroprotective role in CA. In order to further identify the concrete mechanism of microglial activation underlying TGF-β1 neuroprotective role in CA, we applied conditioned medium (CM) from microglial culture supernatants to primary Purkinje neurons *in vitro* and confirmed that inhibiting microglial inflammatory responses was required for TGF-β1 action on Purkinje neurons.

## Materials and Methods

### Experimental Model of CA in Rats

Male adult Sprague-Dawley rats weighing 220-260 g were obtained from the Center of Experimental Animals of Nantong University, China. Experiments were performed in accordance with the policy guidelines of the National Institute of Health Guide for the Care and Use of Laboratory Animals (NIH Publications No. 80-23), revised in 1996. Rats were kept under 12-h light/12-h dark conditions at 22°C with *ad libitum* access to food and water. Induction of CA in rats was achieved by intraperitoneal injection of 3-AP (55 mg/kg of body weight), a neurotoxin that particularly lesions inferior olive neurons in brain stem and eventually leads to ataxia in rats.

### Intracerebroventricular (ICV) Injection of TGF-β1

The experimental rats received one single injection of TGF-β1 (R&D Systems, United States) on day seven after 3-AP infusion. TGF-β1 was unilaterally injected into the lateral ventricle of rats mounted on a stereotaxic frame (David Kopf 902-A, United States). The procedure was carried out under anesthesia by intraperitoneal injection of pentobarbital (55 mg/kg). After exposing the skull and removing the connective tissues, TGF-β1 (25 or 50 ng dissolved in 5 μl saline) was injected into the right lateral ventricle at the following coordinates: −0.8 mm anterioposterior, 1.5 mm mediolateral, and 3.8 mm dorsoventral (Paxinos and Watson, 1998), using the bregma as the zero coordinates. Injections were carried out over 12.5-min period with a constant infusion rate of 0.4 μl/min. Control animals only received same volume of saline solution. Thus, for *in vivo* experiments, the rats were randomly assigned into five groups: control group, 3-AP injection, vehicle (saline, 5 μl) or TGF-β1 (25 or 50 ng in 5 μl) treatment after 3-AP injection. Following ICV injection of TGF-β1, behavior and motor changes were closely observed every day until the rats were sacrificed. On day four following TGF-β1 treatment, some of the detections described below were carried out.

### Behavioral and Motor Coordination Assessments

Behavior and motor coordination were analyzed using the open field and rota-rod tests. For the open field test, rats were put in a square activity chamber (50 cm × 50 cm rectangular box with a wall height of 50 cm). The floor of the chamber was divided into nine identical squares. The equipment was kept in a quiet testing room and cleaned with 70% ethanol before testing of each animal. A video camera over the chamber was installed to record the activities of rats automatically. Rats were carefully placed in the center of the open field. We quantified the locomotor activity by analyzing the number of squares crossed by the rat and speed of movement during a 2-min period. The rota-rod test is a standard test to evaluate motor coordination and balance in rodents and is particularly sensitive in detecting cerebellar dysfunction. Basically, rats were placed on a rotating rod at an accelerating mode (from 4 rpm to 40 rpm during a period of 5 min) in a rota-rod apparatus (Ugo Basile, Italy). The time keeping on the rotating rod was recorded as latency of rat falling, and the average latency time of three consecutive trials was recorded.

### Immunohistochemistry for Glial Cells and Calbindin-Positive Neurons

Detections of glial cells and calbindin-positive neurons were carried out in the brain stem and cerebellum tissues by immunohistochemistry. In detail, anesthetized rats were transcardially perfused with saline followed by 4% paraformaldehyde. The rat brains (containing the cerebrum, cerebellum, and brain stem) were dissected and fixed in 4% paraformaldehyde for 6 h, and then immersed in 30% sucrose in PBS for 48 h, flash-frozen, and mounted in OCT medium (Thermo, United States). Subsequently, 15 μm-thick sections of the brain stem and cerebellum were cut and mounted on glass slides. After 1 h of blocking (10% normal goat serum containing 0.3% Triton X-100 in 0.01 M PBS), the sections were immunostained with mouse anti-CD11b (for microglia, 1:200; Serotec, United Kingdom), anti-GFAP (for astrocytes, 1:400; Serotec, United Kingdom) or anti-calbindin (1:2000; Sigma-Aldrich, United States) for 48 h at 4°C. After rinsing in 0.01 M PBS, the sections were incubated overnight at 4°C in goat secondary antibodies conjugated to FITC (1:200, Jackson, United States) or Alexa Fluor^®^594 (1:400, Jackson, United States), respectively. Finally, images were observed under a fluorescence microscope (Leica, Germany).

### Flow Cytometric Analysis

To measure the effect of TGF-β1 on microglial surface marker expression after 3-AP infusion, flow cytometry was conducted. On day four after TGF-β1 injection, rats were anesthetized with sodium pentobarbital (55 mg/kg) and perfused with cold PBS via the left ventricle of the heart to eliminate cells in the vasculature. Brain stem and cerebellum tissues were isolated, respectively, and digested with collagenase D (2.5 mg/ml; Roche, Penzberg, Germany) and DNaseI (1 mg/ml; Sigma-Aldrich, St. Louis, MO, United States) at 37 °C for 20 min. Then, the single cells were collected by passing the tissues through a 70 μm cell strainer, followed by a percoll gradient (25 and 75%) centrifugation. The mononuclear cells at the 25/75 interphase were obtained for subsequent analysis. The cells were labeled with APC-conjugated anti-CD11b, FITC-conjugated anti-CD40 or PE-conjugated anti-CD86 (all from eBioscience, San Diego, CA, United States) or the appropriate isotype control antibody. The frequency of the respective immunoreactive cells was expressed as a percentage in total mononuclear cells using a FACS Calibur flow cytometer (BD Biosciences, United States) equipped with CellQuest software.

### Primary Culture of Rat Microglial Cells

Primary rat microglial cultures were prepared from cerebral cortices of one-day-old neonatal Sprague-Dawley rats as described previously (Chen et al., 2017) with slight modifications. After removing the meninges, the cortical tissues were digested with 0.25% trypsin-EDTA at 37°C for 30 min, followed by mechanical triturating in DMEM/F12 with 10% fetal bovine serum (FBS). The mixed cortical cells were filtered with a nylon mesh cell strainer (70 μm) and seeded at a density of 5 × 10^5^ cells/cm^2^ on non-coated plastic plates in DMEM/F12 with 10% FBS in 5% CO_2_ at 37°C. The culture medium was changed 24 h later, and then every three to five days thereafter. After achieving confluency at about 12-14 days *in vitro*, the microglia were isolated from mixed glial cultures via shaking the flasks at 220 rpm for 4 h on a rotary shaker. The supernatant containing the detached microglial cells was collected and reseeded for 1 h to allow microglial attachment. After 1 h, the non-adherent cells were removed and the microglia isolated from shaking method were resuspended in fresh medium, and seeded onto the plates precoated with poly-L-lysine for further studies. The obtained microglia purity was approximately 90-95% as verified by CD11b staining (Biolegend, United States).

### Drug Exposure *in vitro*

In the primary microglia-enriched cultures prepared from neonatal rat glia by the shaking, 3-AP was applied at the concentration gradient of 100, 200, 400, and 800 μM on day 10 after the enriched microglia were cultured. After incubation for 72 h, TGF-β1 (10 or 30 ng/ml) was added to the microglial cultures (1 × 10^6^ per well), which were then incubated for 48 h. Finally, we collected the microglia and culture supernatants, and determined microglial activation.

### Preparation of Microglia-Derived CM

The CM was prepared based on the previous method (Li A. et al., 2006). At first, we obtained microglia-enriched cultures as mentioned above. The enriched microglia (1 × 10^6^ per well) were incubated with 1% 3-AP for 72 h at 10 days after the microglia were cultured. Then TGF-β1 (30 ng/ml) or tumor necrosis factor (TNF)-α neutralizing antibody (1 μg/ml) was added to the microglia and cultured for 48 or 6 h, respectively. Then the supernatants of microglia were collected as the CM, which were utilized to culture with the primary seven-day-old Purkinje neurons for 48 h.

### Primary Cerebellar Purkinje Cell Culture

A pregnant Sprague-Dawley rat was purchased from Center of Experimental Animals of Nantong University and newborn rats were euthanized under cryogenic anesthesia on the first day of birth. Briefly, cerebellar cortices were collected from eight new-born rats and rinsed in the Hank’s buffered saline under sterile conditions. After removing the pia mater and superficial blood vessels using micro tweezers, the cerebellar cortices were transferred into serum-free DMEM culture medium. The cerebellar cortices were then minced with sterile scissors and digested with 0.125% trypsin-EDTA solution (Invitrogen, Grand Island, NY, United States) for 15 min with continued shaking at 37°C. Then the enzymatic digestion was terminated by the addition of DMEM culture medium containing 10% FBS. The dissociated cells were suspended in DMEM culture medium and plated at a density of 2.5 × 10^6^/cm^2^ in the wells of 24-well plates precoated with 25 μg/ml poly-L-lysine (Sigma). Then, at 24 h after plating, the culture medium was replaced with serum-free medium and one half of the medium was replaced with fresh medium every two days. The mixed cerebellar cells were cultured in a 5% CO_2_ incubator at 37°C for seven days and the purity of Purkinje cells was verified by calbindin and Hoechst immunohistochemical identification. Subsequently, the microglial CM described above was added to the Purkinje cell culture system and 48 h later, Purkinje cells were collected for further detection.

### Western Blotting Protein Analysis

For *in vivo* experiments, on day four following TGF-β1 administration, the brain stem and cerebellum of all groups were removed and dissected by the method described previously (Chen et al., 2017). Tissue was homogenized (1:10 w/v) with homogenization buffer including 150 mM NaCl, 1% SDS, 50 mM Tris-HCl (pH 7.5), 5 mM EDTA, 4% CHAPS, 0.5% sodium deoxycholate, 1% NP-40, 1% Triton X-100, and 10 μl/ml protease inhibitor cocktail (Sigma-Aldrich), sonicated, and heated to 100°C for 10 min. For *in vitro* experiments, the proteins from cultured cells were extracted with lysis buffer containing 62.5 mM Tris-HCl (pH 6.8), 4% β-mercaptoethanol, 2% SDS, 10% glycerol, 1 mM phenylmethanesulfonyl, and 50 mM DTT. Homogenates were centrifuged at 11,000 *g* for 20 min, and proteins were processed for Western blot analysis to determine the relative levels. The biocinchoninic acid assay (Pierce) was used to determine protein concentrations. Protein samples (45 μg per lane) were separated by 10% sodium dodecyl sulfate-polyacrylamide gel electrophoresis and transferred onto polyvinylidene difluoride membranes (Pall, United States) using an electroblotting apparatus (Bio-Rad, United States). Membranes were soaked in blocking solution (0.1 M PBS and 5% dry skimmed milk, pH 7.4) and incubated with the following primary antibodies diluted in 0.1 M PBS and 1% dry skimmed milk, pH 7.4: rabbit anti-CD11b, rabbit anti-calbindin (all diluted at 1:500; from Abcam, Cambridge, United Kingdom), rabbit anti-caspase-3, and rabbit anti-caspase-9 (all diluted at 1:1000, from Cell Signaling Technology, Danvers, MA, United States). Monoclonal anti-β-actin antibody was used to monitor the amount of protein loading as an internal standard. The membranes were probed at 4°C overnight and then incubated with appropriate fluorescence-conjugated secondary antibodies (1:5000, Rockland Immunochemicals, United States) for 2 h at room temperature. The density of stained bands was scanned and quantified with Odyssey laser scanning system (LI-COR, Lincoln, NE, United States) and the data were normalized in relation to β-actin levels.

### Enzyme-Linked Immunosorbent Assay (ELISA) for Concentrations of Cytokines and Neurotrophic Factors

An ELISA technique was performed to determine the levels of TNF-α, interleukin (IL)-1β, and insulin-like growth factor (IGF)-1 in the brain stem and cerebellum or in the cell culture supernatants. The supernatants of the brain stem or cerebellar homogenates were obtained using RIPA lysis buffer on day four following TGF-β1 administration in rats. The culture supernatants of microglial cells treated with different concentrations of 3-AP were collected. Samples were frozen at −80°C. After thawing, samples were centrifuged at 2000 rpm for 20 min at 4°C, and the concentrations of TNF-α, IL-1β, and IGF-1 were determined using the ELISA kits (eBioscience, United States, for TNF-α and IL-1β, and R&D Systems, United States, for IGF-1) according to the manufacturers’ protocol. Wells of a 96-well polystyrene microtiter plate were precoated with the recombinant antibodies. Samples and standards were incubated with the respective HRP-conjugated IgG polyclonal antibodies. After washing and addition of substrate, the absorbance was read at 450 nm. The standard curve was obtained and the target levels of cytokines and neurotrophic factor in the test samples were calculated.

### Real-Time PCR Analysis

Real-time PCR analysis was utilized to detect the relative gene expression levels of cytokines, TNF-α, IL-1β, and neurotrophic factor IGF-1. For *in vivo* experiments, the brain stem and the cerebellum were removed rapidly from decapitated rats on day four following TGF-β1 treatment and cooled immediately in ice-cold buffer (pH 7.4) containing 126 mM NaCl, 1.25 mM NaH_2_PO_4_, 5 mM KCl, 2 mM CaCl_2_, 25 mM NaHCO_3_, 10 mM D-glucose, and 2 mM MgSO_4_. For *in vitro* experiments, the microglial cells were harvested at 48 h after TGF-β1 application. Total RNA was prepared from brain tissues (the brain stem or cerebellum) or cultured microglia using TRIzol reagent (Invitrogen) and purified with the RNeasy mini kit (Qiagen, United States) prior to complementary DNA (cDNA) synthesis. The real-time quantitative PCR was performed on cDNA by using the Rotor-Gene 3000 Realtime Cycler (Corbett Research, Australia) with SYBR green I as the detection system. Each 20 μl of reaction mixture contained 2 μl cDNA, 2 μl PCR buffer, 2.5 mM MgCl_2_, 0.15 mM of each dNTP, 0.15 μM of each pair of oligonucleotide primers, and 1 μ Taq DNA polymerase. The reaction procedures were as follows: an initial step at 95°C for 10 min, 40 cycles of 95°C for 15 s, 62°C for 20 s, and 72°C for 20 s. Signals were analyzed using Rotor-Gene 6.0 software and the expression levels of target genes were determined using the 2^–ΔΔ*Ct*^ method. β-actin was utilized as an internal reference. The primer sequences were listed as follows: *Tnf*α (5′-CCACCACG CTCTTCTGTCTAC-3′ and 5′-ATCTGAGTGTGGGGTCT GG-3′); *Il1*β (5′-CTT CCTTGTGCAAGTGTCTG-3′ and 5′-CA GGTCATTCTCATCACTGTC-3′); *Igf1* (5′-TTTTACTTCAACA AGCCCACA-3′ and 5′-CATCCACAATGCCCGTCT-3′); β*-actin* (5′-CGTTGACATCCGTAAAGACC-3′ and 5′-TAGAGCCACC AATCC ACAC-3′).

### Immunocytochemistry

The CD11b immunocytochemistry was performed to identify the morphologic changes of primary microglia cultures treated with TGF-β1 in the presence of 3-AP. Briefly, the paraformaldehyde-fixed cultures were sequentially incubated with blocking solution for 30 min, primary antibody to CD11b (Serotec, United Kingdom; 1:200) overnight at 4°C, and fluorescent secondary antibody conjugated to FITC (1:500, Jackson, United States) for 6 h at room temperature. The CD11b immunoreactive cells were observed under a fluorescence microscope (Leica, Germany).

The Purkinje cells cultured with different CM mentioned above were seeded onto cover slips precoated with poly-L-lysine and fixed in 4% paraformaldehyde for 15 min at room temperature, and then blocked with 0.3% Triton X-100 and 3% goat serum in 0.01 M PBS (pH 7.3) for 30 min. Subsequently, the cells were incubated with primary antibody mouse anti-calbindin at 1:1000 (Sigma-Aldrich, United States) overnight at room temperature, followed by incubation with Alexa Fluor^®^594 conjugated goat anti-mouse IgG antibody (1:500, Jackson, United States) for 4 h at room temperature. To assess apoptosis, terminal deoxynucleotidyl transferase-mediated deoxyuridine triphosphate-biotin nick end labeling (TUNEL) staining was carried out using the *in situ* Cell Death Detection Kit (Roche Applied Science, Mannheim, Germany) according to the manufacturer’s instructions. In calculating the apoptotic index, a total of five random visual fields in each coverslip were counted for TUNEL-stained and calbindin-stained cells.

### Statistical Analysis

Data were presented as the means ± standard deviation (M ± SD) of each group. Software used in our analyses was the Statistics Package for Social Science (SPSS, 12.0, SPSS, Chicago, IL, United States). The differences between means were determined by the one-way analysis of variance (ANOVA) followed by the Student-Newman-Keul’s test for pairwise comparisons of the data. Differences of *p* < 0.05 were considered as statistically significant.

## Results

### TGF-β1 Ameliorates 3-AP-Induced Behavioral and Motor Coordination Impairments

**FIGURE 1:**
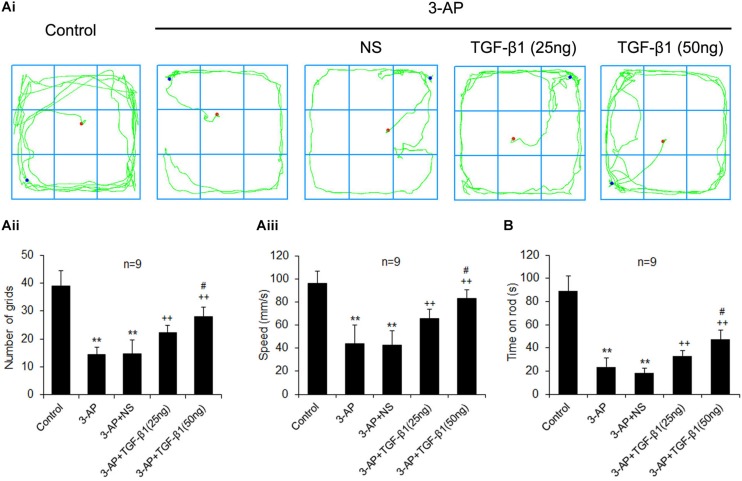
TGF-β1 ICV injection in rats reduces 3-AP-induced impairments in behavior and motor coordination. TGF-β1 (25 or 50 ng in 5 μl) was given ICV on day seven after 3-AP intraperitoneal injection in rats. The open field **(A)** and rota-rod **(B)** tests were performed to evaluate locomotor activity and motor coordination of rats on day four following TGF-β1 treatment, respectively. **(Ai)** illustrates the represented movement traces of each group. The statistical histograms in **(Aii)** and **(Aiii)** show the number of moving through grids and the speed of movement in open field test, respectively. The statistical histogram in **(B)** represents the time keeping on the rota rod of each group. Error bars indicate standard deviation of the mean. One-way ANOVA, followed by the Student-Newman-Keul’s test. **p < 0.01, versus control rats; ++ *p* < 0.01, versus 3-AP-treated rats; #*p* < 0.05, versus 3-AP + TGF-β1 (25 ng/5 μl)-treated rats.

Transforming growth factor-β1 ICV injection was performed seven days after 3-AP administration. To assess whether TGF-β1 administration improved behavior and motor coordination after 3-AP injection, the open field and rota-rod tests were performed, respectively, on day four following TGF-β1 administration. In open field test, 3-AP treatment alone remarkably reduced the number of moving through grids and the speed of movement with respect to the control rats (Figure 1A). Importantly, compared with 3-AP treatment alone, TGF-β1 ICV administration (25 or 50 ng in 5 μl) after 3-AP invasion significantly elevated the number of moving through grids and the speed of movement, and the effect of TGF-β1 (50 ng in 5 μl) administration was more obvious (Figure 1A). Nevertheless, ICV treatment of the same volume of saline after 3-AP invasion did not significantly alter the above changes induced by 3-AP (Figure 1A). Similarly, in rota-rod test, 3-AP treatment alone decreased the time of rats keeping on the rota-rod relative to the control rats (Figure 1B). TGF-β1 ICV administration (25 or 50 ng in 5 μl) after 3-AP invasion lengthened the time compared with 3-AP treatment alone, and also the treatment with TGF-β1 (50 ng in 5 μl) was more effective (Figure 1B). These results showed that TGF-β1 improved behavior and motor coordination in 3-AP ataxic rats.

### TGF-β1 Prevents 3-AP-Induced Neuronal Loss and Apoptosis in the Cerebellum and the Brain Stem of CA Rats

**FIGURE 2:**
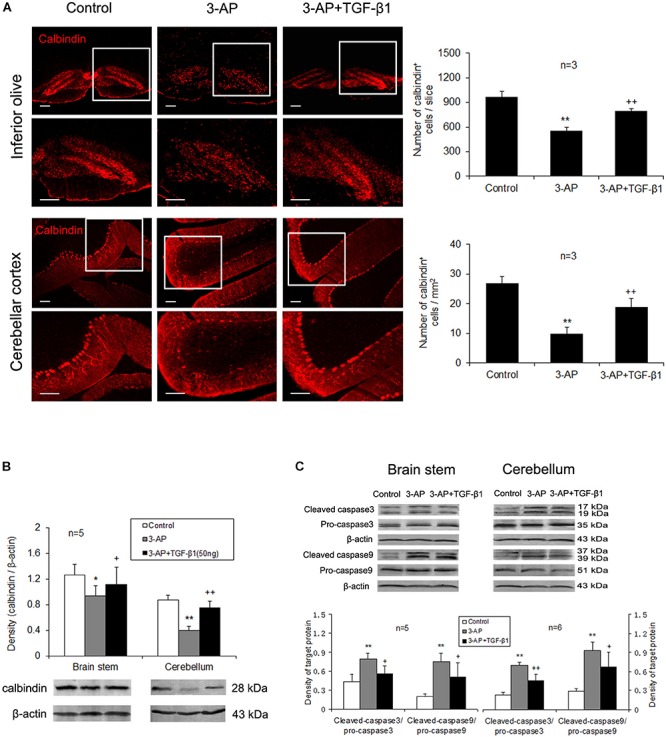
TGF-β1 treatment ameliorates 3-AP-induced calbindin-positive neuronal loss and apoptosis in the brain stem and the cerebellum. TGF-β1 (50 ng in 5 μl) was given with the ICV at 7 days following 3-AP administration. On day 4 after TGF-β1 treatment, calbindin immunohistochemistry **(A)** and Western blot analyses for calbindin **(B)** and pro-apoptotic enzymes, caspase-3 and caspase-9 **(C)** were performed. These experimental findings show that 3-AP induces decreases in number of calbindin-positive neurons **(A)** and calbindin protein level **(B)** and increases in the expressions of caspase-3 and caspase-9 **(C)** both in the inferior olive and in the cerebellar cortex. TGF-β1 (50 ng in 5 μl) treatment after 3-AP injection prevented all these changes caused by 3-AP **(A-C)**. Scale bar = 100 μm. Error bars indicate standard deviation of the mean. One-way ANOVA, followed by the Student-Newman-Keul’s test. *p < 0.05, **p < 0.01, versus control rats; + *p* < 0.05, ++ *p* < 0.01, versus 3-AP-treated rats.

Calbindin is a calcium binding protein expressed by a population of neurons in the inferior olive as well as the cell bodies and terminals of Purkinje cells in the cerebellum. To reveal TGF-β1 neuroprotection in CA rats, we detected the calbindin-positive neurons in the brain stem and cerebellum. A single injection of 3-AP significantly decreased the number of calbindin-positive neurons in the inferior olive and the cerebellar cortex verified by immunohistochemistry (Figure 2A). TGF-β1 (50 ng in 5 μl) administration after 3-AP invasion partially alleviated 3-AP-induced reduction of calbindin-positive neurons (Figure 2A). Meanwhile, a similar result was found in calbindin protein expression in the inferior olive and cerebellar cortex (Figure 2B). To further confirm TGF-β1 neuroprotection against 3-AP toxicity, we measured the expressions of apoptosis-related genes, caspase 3 and caspase 9 in the brain stem and cerebellum. Our findings exhibited that 3-AP treatment caused significant increases in the ratio of cleaved caspase 3 to pro-caspase 3 and cleaved caspase 9 to pro-caspase 9 either in the brain stem or in the cerebellum when compared with the control rats, and TGF-β1 (50 ng in 5 μl) treatment after 3-AP injection obviously reduced the ratio increases induced by 3-AP (Figure 2C). These findings indicated TGF-β1 alleviated 3-AP-induced calbindin-positive neuronal loss and apoptosis in the brain stem and cerebellum.

### TGF-β1 Attenuates 3-AP-Induced Microglial Activation and Inflammatory Responses in CA Rats

**FIGURE 3:**
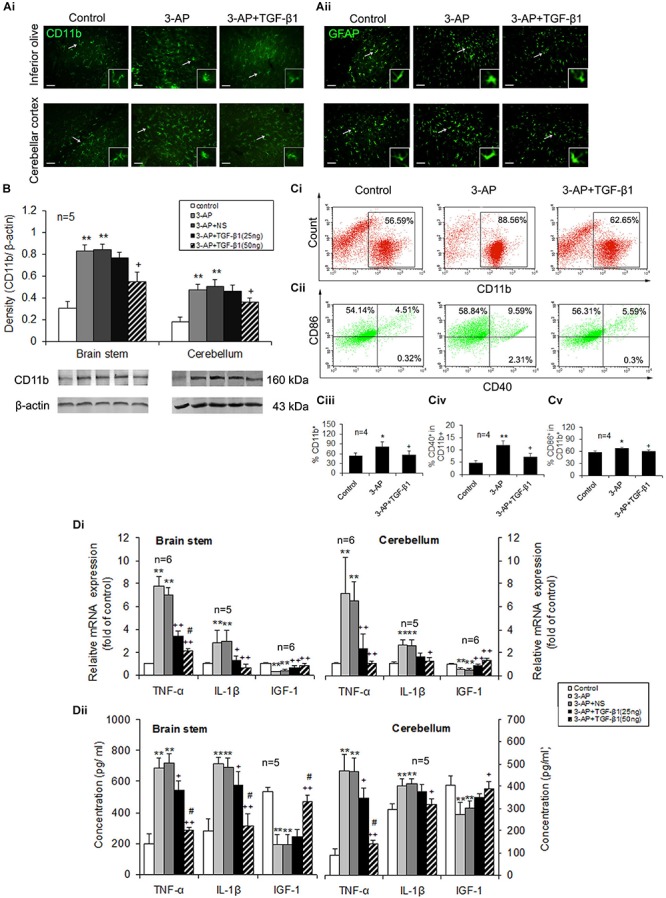
TGF-β1 suppresses 3-AP-induced glial activation and inflammatory responses. TGF-β1 was ICV injected at seven days following 3-AP injection in rats. On day four after the TGF-β1 administration, microglia and astrocytes in the brain stem or the cerebellum were evaluated for their activation or function. **(A)** Immunofluorescent images of CD11b and GFAP staining in the inferior olive and the cerebellum. Scale bar = 50 μm. **(B)** Western blot analysis of CD11b expression level in the brain stem or the cerebellum. **(C)** Representative fluorescent dot images showing the percentage of CD11b^+^ cells as well as percentages of CD40^+^ or CD86^+^ cells in CD11b^+^ cells. Microglial cells from adult rat brain stem or cerebellum were isolated using Percoll density gradient centrifugation and analyzed using a flow cytometer. The microglial cells were stained with the cell surface markers, CD11b, CD40, and CD86. In **(Ci)**, the numbers in the boxes represent the percentage of CD11b^+^ cells. In **(Cii)**, there are four plots (UL, upper left; UR, upper right; LL, low left; and LR, low right) in a fluorescent dot image of each treatment, and the numbers in the plots indicate percentages. The sum of the two numbers in UL and UR plots represents the percentage of CD86^+^ cells in CD11b^+^ cells, and CD40^+^ cell percentage in CD11b^+^ cells is the sum of UR + LR. Statistical graphs **(Ciii-Cv)** show differences in expression of the three cell surface markers of microglia between the different treatments. **(Di)** Real-time PCR analysis for gene expression levels of *Tnf*α, *Il1*β and *Igf1* in the brain stem and the cerebellum. **(Dii)** ELISA analysis for contents of TNF-α, IL-1β and IGF-1 in the brain stem and the cerebellum. Error bars indicate standard deviation of the mean. One-way ANOVA, followed by the Student-Newman-Keul’s test. *p < 0.05, **p < 0.01, versus control rats; + *p* < 0.05, ++ *p* < 0.01, versus 3-AP-treated rats; #*p* < 0.05, versus 3-AP + TGF-β1 (25 ng/5 μl)-treated rats.

To figure out that TGF-β1 neuroprotection against 3-AP toxicity correlated with its alleviation of neuroinflammation in CA rats, we mainly examined microglial activation and inflammatory responses in the brain stem and cerebellum. The CD11b immunohistochemical images showed that microglia in the brain stem and cerebellum of 3-AP-treated rats were larger in soma size than that in control rats, which displayed a morphologic change of microglial activation, and TGF-β1 (50 ng in 5 μl) administration lessened the increased soma size and the retracted processes of the CD11b-stained cells induced by 3-AP (Figure 3Ai). In addition, TGF-β1 treatment also inhibited 3-AP-induced astrogliosis in the brain stem and cerebellum, and the morphologic alteration in GFAP-stained astrocytes was illustrated in Figure 3Aii. Similarly, 3-AP treatment enhanced CD11b protein expression level both in the brain stem and the cerebellum, and TGF-β1 (50 ng not 25 ng in 5 μl) treatment notably prevented 3-AP-induced elevation of CD11b expression (Figure 3B). To further confirm the inhibition of microglial activation by TGF-β1, we isolated the brain microglia in the cerebellum and brain stem to examine the expression of the cell surface markers (CD11b, CD40, and CD86) by flow cytometry. CD11b-positive cells that represent microglia were more than 50% in the collected cells prepared from adult rat cerebellum and brain stem by the percoll density separation. The percentage of CD11b-positive cells was significantly increased in 3-AP-treated rats opposed to the control rats, meanwhile the percentages of CD40^+^ or CD86^+^ cells in CD11b^+^ cells were also increased in the cerebellum and brain stem by 3-AP exposure (Figure 3C). Significantly, administration with TGF-β1 (50 ng in 5 μl) prevented the increased percentages of the cells expressing the above surface markers induced by 3-AP (Figure 3C). Furthermore, compared to the control rats, 3-AP treatment resulted in a prominent increase of the pro-inflammatory cytokines, TNF-α and IL-1β, as well as a remarkable decrease of the neurotrophic factor, IGF-1, in the mRNA (Figure 3Di) and protein (Figure 3Dii) levels both in the cerebellum and the brain stem. The mRNA levels of TNF-α and IGF-1 and protein level of TNF-α in the cerebellum were decreased after TGF-β1 treatment with 25 ng/5 μl. Meanwhile, TGF-β1 (25 ng/5 μl) treatment reduced all the cytokine levels except IGF-1 protein level in the brain stem (Figure 3D). Significantly, ICV injection of TGF-β1 (50 ng in 5 μl) prevented all the cytokine changes induced by 3-AP (Figure 3D).

### TGF-β1 Inhibits 3-AP-Elicited Activation and Inflammatory Responses in Primary Cultured Microglia

**FIGURE 4:**
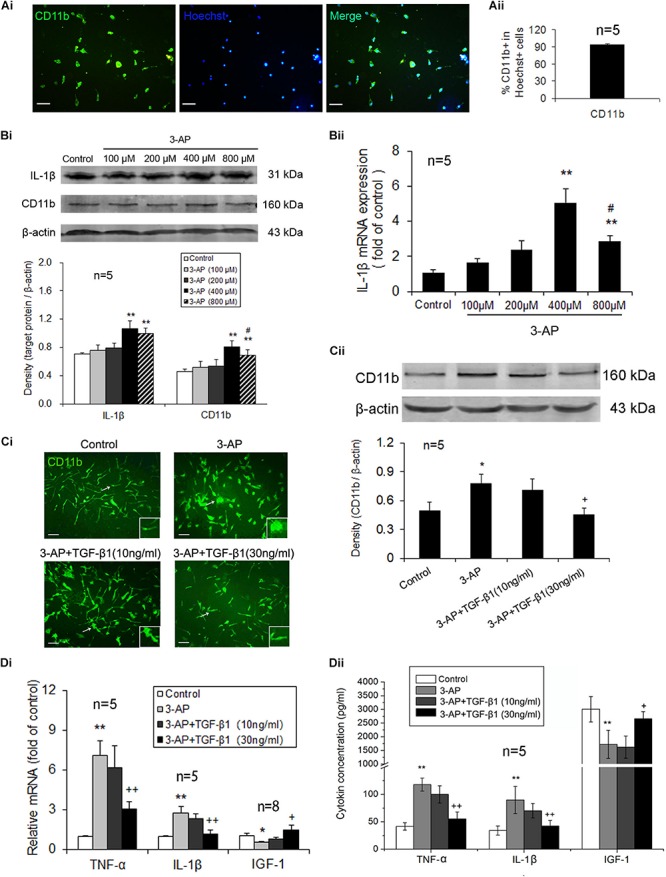
TGF-β1 treatment suppresses 3-AP-induced microglial activation and inflammatory responses *in vitro*. After the microglia that had been isolated from neonatal rat glia by the shaking were incubated for ten days, different concentrations of 3-AP were added to the microglial cultures 72 h prior to TGF-β1 (10 or 30 ng/ml) application, which were incubated for 48 h. **(A)** The purity identification of primary cultured microglia by CD11b and Hoechst immunohistochemistry. **(B)** Gene and protein expression levels of CD11b and IL-1β in the primary microglial cultures treated with different concentrations of 3-AP by real-time PCR and Western blot analyses. **(Ci)** CD11b immunostaining in the primary microglial cultures treated with TGF-β1 (10 or 30 ng/ml) in the presence of 400 μM 3-AP. **(Cii)** Protein expression level of CD11b after TGF-β1 (10 or 30 ng/ml) was applied to the microglial cultures activated by 400 μM 3-AP *in vitro*. **(Di)** Gene expression levels of proinflammatory cytokines *(Tnf*α and *Il1*β) and neurotrophic factor (*Igf1)* in microglia by real-time PCR. **(Dii)** ELISA analyses for concentrations of TNF-α, IL-1β and IGF-1 in microglial culture supernatants. Scale bar = 50 μm. Error bars indicate standard deviation of the mean. One-way ANOVA, followed by the Student-Newman-Keul’s test. *p < 0.05, **p < 0.01, versus control cells; #*p* < 0.05, versus 3-AP (400 μM)-treated cells; + *p* < 0.05, + + *p* < 0.01, versus 3-AP (400 μM) treatment alone.

To further demonstrate the inhibitory effects of TGF-β1 on microglial activation and inflammatory responses, we applied different concentrations of TGF-β1 to primary cultured microglial cells activated by certain concentration of 3-AP and then observed the changes of CD11b expression level as well as the mRNA and protein levels of the inflammatory mediators, TNF-α and IL-1β, and the neurotrophic factor IGF-1. The purity of primary cultured microglial cells prepared from neonatal rat glia by the shaking was more than 90% by CD11b and Hoechst immunohistochemical identification (Figure 4A). Primary cultured microglia cells were treated with 3-AP in different concentrations (100, 200, 400, and 800 μM) for 72 h. As shown in Figure 4B, 3-AP treatment upregulated the protein expression levels of CD11b and IL-1β as well as the mRNA level of IL-1β in a concentration-dependent manner, especially in 3-AP treatment with 400 μM. Therefore, in the later experiments, we employed TGF-β1 (10 or 30 ng/ml) into primary cultured microglia activated by 400 μM of 3-AP. The CD11b immunocytochemistry showed that 3-AP-treated cells were larger in soma size and retracted in processes than control cells and TGF-β1 (especially 30 ng/ml) reduced the increased soma size and the retracted processes of CD11b-stained cells induced by 3-AP (Figure 4Ci). In consist with the morphologic changes, 3-AP (400 μM) treatment increased CD11b expression level in microglial cultures and TGF-β1 (30 ng/ml not 10 ng/ml) treatment prevented the increase of CD11b expression caused by 3-AP (Figure 4Cii). Simultaneously, 3-AP treatment with 400 μM induced an upregulation of the inflammatory mediators, TNF-α and IL-1β, and a downregulation of the neurotrophic factor IGF-1 in the microglial cultures (Figure 4D). TGF-β1 (30 ng/ml not 10 ng/ml) application in microglia prevented all these changes caused by 3-AP (Figure 4D).

### TGF-β1 Attenuates 3-AP-Induced Apoptosis of Purkinje Neurons by Inhibiting the Release of TNF-α From Microglial Cells

**FIGURE 5:**
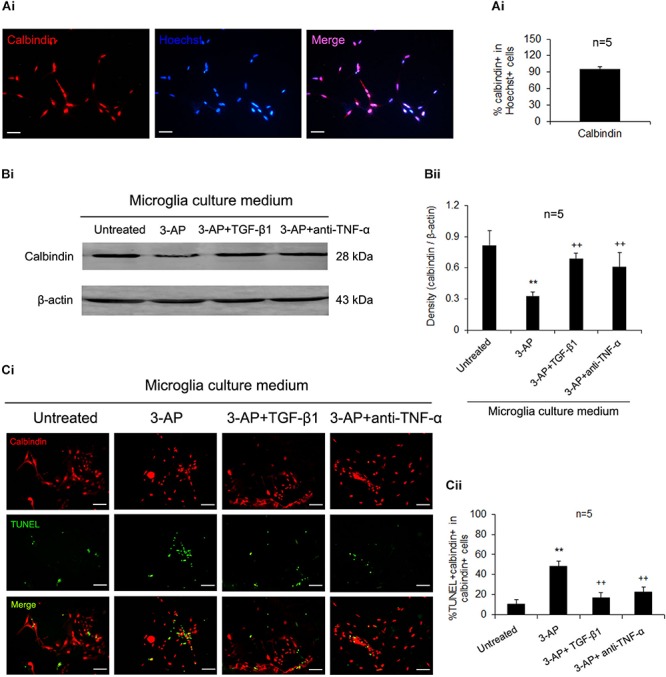
TGF-β1 neuroprotection against 3-AP toxicity is achieved by its inhibition of microglial releasing TNF-α. The seven-day-cultured recipient Purkinje neurons were incubated with the CM derived from donor microglia cultures treated with 1% 3-AP, or co-treated with 3-AP and TGF-β1 (30 ng/ml) or 3-AP and TNF-α neutralizing antibody (1 μg/ml) for 48 h. The purity of primary Purkinje cells was identified by calbindin and Hoechst immunohistochemistry **(A)**. The Purkinje neurons were then quantified by Western blot densitometry for expression of calbindin **(B)** and doubly stained with calbindin and TUNEL fluorescent immunocytochemistry **(C)**. The statistical data (M ± SD) show that 3-AP-treated CM decreases calbindin expression level and increases the number of calbindin-TUNEL double positive cells, and the 3-AP plus TGF-β1-administered CM or the 3-AP plus TNF-α neutralizing antibody -administered CM relieve the changes caused by 3-AP-stimulated CM. Scale bar = 50 μm. One-way ANOVA, followed by the Student-Newman-Keul’s test. **p < 0.01, versus untreated CM; ++ *p* < 0.01, versus 3-AP-treated CM.

In order to investigate the neuroprotective mechanism of TGF-β1 in CA, the primary culture of Purkinje neurons was carried out and various components of CM from microglial cultures treated with 3-AP alone, 3-AP plus TGF-β1 or 3-AP plus TNF-α neutralizing antibody were performed to culture recipient Purkinje neurons. Cultures were maintained for 48 h and then determined for calbindin protein expression and apoptosis of Purkinje cells. The purity of primary cultured Purkinje neurons prepared from neonatal rat was more than 95% by calbindin and Hoechst immunohistochemical identification (Figure 5A). As shown in Figure 5B, the treatment with CM derived from 3-AP-stimulated microglial cultures decreased calbindin expression level in recipient Purkinje cell cultures when compared with the treatment with CM derived from unstimulated microglial cultures. Compared with that of 3-AP-stimulated CM, calbindin expression levels were significantly increased in Purkinje neuron cultures when exposed to 3-AP plus TGF-β1-treated CM or 3-AP plus TNF-α neutralizing antibody-treated CM (Figure 5B). Furthermore, the apoptosis of Purkinje neurons was detected by immunocytochemical staining. As shown in Figure 5C, the number of calbindin-TUNEL double positive cells was markedly increased in the CM of microglia treated with 3-AP. In contrast, with respect to that in 3-AP-stimulated CM, there were notable decreases in the number of calbindin-TUNEL double positive cells in the CM of 3-AP plus TGF-β1-treated and 3-AP plus TNF-α neutralizing antibody-treated microglial cultures (Figure 5C). These findings suggested that TGF-β1 neuroprotection against 3-AP toxicity was achieved partly by inhibiting the release of TNF-α from microglia.

## Discussion

Cerebellar ataxia is considered to be a range of brain disorders characterized by lack of motor coordination induced by disturbances in the cerebellum and its associated circuits (Marmolino and Manto, 2010). 3-AP, known as methyl β-pyridyl ketone, is a metabolic antagonist utilized to decrease nicotinamide level of laboratory animals in research. Although a large number of laboratory animals with neurological mutations have been reported and numerous relevant animal models mimicking the phenotype of CAs are becoming available (Manto and Marmolino, 2009), the administration of 3-AP has still been suggested as a classic method to induce the CA animal model and provokes many characteristics similar to the typical features of CA in human (Janahmadi et al., 2009; Wecker et al., 2017). The application of 3-AP selectively lesions the neurons in the medial inferior olive, leading to a loss of climbing fibers innervating cerebellar Purkinje cells and a consequent ataxia manifested by alterations in both balance and gait (Rondi-Reig et al., 1997; Gasbarri et al., 2003; Wecker et al., 2013). Therefore, the present results showing the impairments in behavior and motor coordination along with the increased loss and apoptosis of calbindin-positive neurons in the brain stem and cerebellum following an 3-AP injection, strongly demonstrate that CA-associated neurodegeneration is successfully established. Importantly, TGF-β1 ICV administration ameliorates 3-AP-induced behavioral and motor coordination impairments as well as the neuronal loss and apoptosis in the cerebellum and brain stem. These results suggest that TGF-β1 alleviates the CA-associated neurodegeneration and plays a neuroprotective role in CA behavior and pathology. However, in our present study the effect of TGF-β1 on motor coordination of 3-AP-treated rats was limited, which might correlate with the existence of damage of other brain regions (such as motor cortex) caused by 3-AP treatment as well as the dose and effective maintenance time of TGF-β1 single injection *in vivo* in our experiment.

Transforming growth factor-β1 is a pleiotropic cytokine with immunosuppressive and anti-inflammatory properties (Le et al., 2005; Vivien and Ali, 2006). TGF-β1 is also known to be upregulated in the brain in most neurodegenerative diseases, such as PD, AD, MS, and ALS, and slow down the neurodegenerative process by preventing tissue damage and neural apoptotic death (Mogi et al., 1994; Wyss-Coray et al., 1997; Ilzecka et al., 2002; Zetterberg et al., 2004; Mirshafiey and Mohsenzadegan, 2009) so as to keep the homeostasis during disease progression. These facts indicate that TGF-β1 plays a potential neuroprotective role in neurodegenerative diseases (Martínez-Canabal, 2015). Neurons express little if any TGF-β1 less in normal states (da Cunha et al., 1993; Ata et al., 1997; Vivien et al., 1998; De Groot et al., 1999; de Sampaioe Spohr et al., 2002), but throughout the CNS, the isoforms of TGF-β and TGF-β receptors have been found in all glial cells, including astrocytes and microglia (Vivien et al., 1998; Vivien and Ali, 2006). Recently, several lines of evidence from our laboratory have shown that TGF-β1 exerts neuroprotective role in neurodegenerative diseases by inhibiting microglia-mediated neuroinflammation (Shen et al., 2014; Chen et al., 2015, 2017; Liu et al., 2016; Fang et al., 2018), but its role in CA has still not been elucidated. In the present study, apart from generating destruction to neurons, 3-AP induced an excessive activation of microglial cells as determined by morphological alteration, the upregulated CD11b expression and percentage of CD40^+^ and CD86^+^ microglial cells, as well as the enhanced inflammatory responses manifested as the increased mRNA and protein level of the pro-inflammatory mediators (TNF-α and IL-1β) and decreased level of the neurotrophic factor IGF-1 in both the brain stem and the cerebellum. These data assume that 3-AP-elicited neuronal dysfunctions trigger microglial activation and subsequently these activated microglia secrete pro-inflammatory cytokines to further promote neuronal damage and death. Significantly, TGF-β1 treatment notably attenuated 3-AP-induced microglial activation and inflammatory responses in CA rats. These results *in vivo* strongly indicate that TGF-β1 reduction of 3-AP-induced microglial activation and inflammatory responses may be a vital mechanism underlying its neuroprotection in CA rats. In addition, TGF-β1 also inhibited 3-AP-induced astrogliosis, which further facilitated the inhibitory role of TGF-β1 in inflammatory responses in CA rats. Except for the activation of endogenous glial cells, the peripheral immune cell infiltration often participates in the neuroinflammatory process in many neurodegenerative diseases. However, the immune cell infiltration in CA has been less reported and we found no experimental evidence for peripheral immune cell infiltration in 3-AP-induced CA in our present study. Therefore, we propose that TGF-β1 might exert its neuroprotection against neurodegeneration in 3-AP-induced CA mainly via inhibition of microglia-mediated neuroinflammation.

In order to better understand the role of TGF-β1 in regulating microglia-mediated neurotoxicity in CA rats, TGF-β1 was directly applied to primary microglial cultures that had been treated with 3-AP *in vitro*. As expected, 3-AP treatment resulted in morphologic changes and increases in CD11b protein expression and the mRNA and protein levels of IL-1β in a concentration-dependent manner in primary microglial cultures, which implying that in addition to initiating microglial activation by triggering neurodegeneration, 3-AP can also directly induce microglial activation. Moreover, we observed the upregulation of the proinflammatory mediators, TNF-α, IL-1β and downregulation of the neurotrophic factor IGF-1 in the 3-AP-treated microglial cultures in the present study. Since the proinflammatory mediators and neurotrophic factors are produced primarily by microglia in the brain, these findings further confirm the fact that 3-AP directly induces microglial activation. Importantly, TGF-β1 administration in microglial cultures inhibited all the above changes caused by 3-AP, which provide a direct evidence for TGF-β1 inhibitory modulation in 3-AP-induced microglial activation. In line with this, the research results from our and other laboratories support the viewpoint that microglial activity *in vitro* can be suppressed by TGF-β1 via decreasing inflammatory mediator (such as TNF-α, IL-1β, NO and ROS) or increasing neurotrophic factor (IGF-1, GDNF, and BDNF) productions induced by LPS, MPP^+^ or Aβ_1__–__42_ (Herrera-Molina and von Bernhardi, 2005; Ramírez et al., 2005; Spittau et al., 2013; Liu et al., 2016; Chen et al., 2017; Fang et al., 2018).

In general, a major feature of neuroinflammation is activation of microglia and astrocytes, with subsequent production of proinflammatory cytokines and chemokines that recruit more neurotoxic glial cells and lead to further neuroinflammation and neuronal damage. For better identifying the detailed mechanism underlying microglial activation in TGF-β1 neuroprotective role in CA rats, the supernatants of microglial cultures treated by 3-AP, 3-AP plus TGF-β1 or 3-AP plus TNF-α neutralizing antibody were performed to primary Purkinje neurons. The microglial CM treated with 3-AP alone potentiated Purkinje cell death as determined by decreased calbindin expression and increased number of calbindin-TUNEL double positive cells, however, the microglial CM treated with 3-AP plus TGF-β1 reduced Purkinje cell death, which was similar to those treated with 3-AP plus TNF-α neutralizing antibody. These data demonstrate that TNF-α may be a vital mediator that transmits 3-AP inflammatory information from microglia to Purkinje neurons and TGF-β1 alleviates Purkinje cell death at least partly by inhibiting microglial release of TNF-α. There is a convincing body of evidence that TGF-β1 inhibits microglial activation and induces downregulation of proinflammatory cytokines in microglia (von Zahn et al., 1997; Basu et al., 2002; Milner and Campbell, 2003; Herrera-Molina and von Bernhardi, 2005; Taylor et al., 2017). Our present results extend the findings and indicate that proinflammatory cytokines, particularly TNF-α released from microglia, mediate TGF-β1 potential protection to cerebellar Purkinje neurons in CA rats.

In summary, 3-AP toxin application leads to behavioral impairment and neuronal loss and apoptosis in the brain stem and cerebellum of CA rats. These changes are accompanied by microglial activation and inflammatory responses. *In vivo*, TGF-β1 ICV administration after 3-AP injection inhibits the neuroinflammatory responses and alleviates behavioral impairments and neurodegeneration. *In vitro*, TGF-β1 directly ameliorates 3-AP-induced microglial activation and diminishes Purkinje cell loss and apoptosis by inhibiting the release of TNF-α from microglial cells. These findings suggest that TGF-β1 plays a neuroprotective role in 3-AP-induced CA via inhibition of microglial activation and at least partly TNF-α release from microglial cells.

## Data Availability Statement

All datasets generated for this study are included in the article.

## Ethics Statement

All of the experiments were performed in accordance with the National Institute of Health Guide for the Care and Use of Laboratory Animals and were reviewed and approved by the Institutional Animal Care and Use Committee of Nantong University.

## Author Contributions

Y-HQ and Y-PP designed the study. B-BC, X-XZ, C-YD, and ZL performed the study. B-BC, X-XZ, Y-HQ and Y-PP analyzed the data. B-BC wrote and Y-PP edited the manuscript. All authors reviewed the final version of the manuscript.

## Conflict of Interest

The authors declare that the research was conducted in the absence of any commercial or financial relationships that could be construed as a potential conflict of interest.
